# Effect of carvedilol versus nebivolol on insulin resistance among non-diabetic, non-ischemic cardiomyopathy with heart failure

**DOI:** 10.1186/s43044-020-00099-5

**Published:** 2020-09-29

**Authors:** Yasser Gaber Metwally, Heba Kamal Sedrak, Inass Fahiem Shaltout

**Affiliations:** 1 Department of Cardiology, Faculty of Medicine, Zagazig University, Zagazig, Egypt; 2 Department of Internal Medicine, Faculty of Medicine, Cairo University, Cairo, Egypt

**Keywords:** Carvedilol, Nebivolol, Insulin resistance, Heart failure

## Abstract

**Background:**

Although B-blockers provide unequivocal benefits in heart failure (HF) management, some B-blockers worsen insulin resistance. It will be a promising strategy to recruit such a B blocker that did not worsen or can even improve insulin resistance (IR).

So, this study aimed to assess the effect of two of the third-generation B-blockers (carvedilol versus nebivolol) on insulin sensitivity state in non-diabetic patients with non-ischemic cardiomyopathy with heart failure.

**Results:**

Out of 43 patients enrolled, 58.1% represented the carvedilol group while 41.9% represented the nebivolol group. Nebivolol improves insulin resistance-related variables (fasting glucose, fasting insulin, and HOMA-IR; *P* < 0.001, 0.01, and 0.01 respectively). The percentage of change at homeostasis model of assessment (HOMA-IR), indicative of insulin sensitivity status, between baseline versus at 3-months follow-up level of intra-group comparison was increased by 4.58% in the carvedilol arm whereas it was decreased by 11.67% in the nebivolol arm, and the difference on the intragroup level of comparison was significant (*P* < 0.001 and 0.01 respectively).

**Conclusion:**

Nebivolol improves insulin resistance-related variables .Nebivolol may be recommended as the B blocker of the first choice for those with non-ischemic cardiomyopathy heart failure with evident insulin resistance; however, larger scaled prospective multicenter randomized trials are needed for confirming our favorable results.

## Background

Myocardial systolic dysfunction is associated with sympathetic hyperactivity evidenced by increased plasma norepinephrine (NE) level, central sympathetic outflow, and NE plasma spillover [1]. Measurement of cardiac NE plasma release using isotope dilution method indicates that in untreated heart failure patients, cardiac NE spillover is increased as much as 50 times similar to levels seen in healthy hearts during maximal exercise [2]. However, in contrast to increased muscle sympathetic nerve activity and NE spillover, patients with heart failure with reduced ejection fraction (HFrEF) may have decreased NE concentration inside the cardiac cells, together with a reduction of post-synaptic beta-receptor density [3]. However, it should be realized that plasma NE level does not necessarily reflect the sympathetic activity level in skeletal muscle [4]. The neurohormonal hyperactivity associating HfrEF represents a compensatory mechanism to maintain cardiac output. The neuronal limb of such response is represented by the sympathetic nervous system (SNS), whereas the humoral limb is represented by the renin-angiotensin aldosterone axis [5]. The overactivation of the sympathetic nervous system in congestive heart failure (CHF) patients is thought to contribute to hyperinsulinism and insulin resistance (IR) [6]. A moderate increase in plasma NE has been observed to reduce glucose tolerance, and insulin sensitivity may be accomplished by increased lipolysis and free fatty acid levels [7].

Moreover, a small increase in plasma NE has been reported to increase fasting blood glucose through transient stimulation of basal hepatic glucose output without altering basal glucose utilization, insulin, or glucagon secretion [8]. On the other hand, insulin does also stimulate the SNS. According to animal and human studies, it has been demonstrated that short-term insulin infusion stimulates the SNS activity [9]. It is evident that acute physiological as well as pharmacological euglycemic hyperinsulinemia increase plasma catecholamine concentration [10]. Furthermore, it is documented that hypertensive patients show an enhanced SNS activity in response to insulin [11]. Hyperinsulinemia may therefore also influence adrenergic activity, contributing to further insulin-resistance worsening (vicious circle).

Evident data suggest that heart failure may not only precipitate insulin resistance but also lead to IR worsening [12]. Although B-blockers provide unequivocal benefits in heart failure management including improving survival, some B-blockers worsen insulin resistance [13, 14]. However, it will be a promising strategy in heart failure therapy to recruit such a B blocker that did not worsen or can even improve insulin resistance. We hypothesized that nebivolol could have a better effect on insulin resistance than carvedilol among non-diabetic, non-ischemic cardiomyopathy with heart failure. Accordingly, this study aimed to investigate the effect of carvedilol versus nebivolol on insulin resistance among non-diabetic, non-ischemic cardiomyopathy with heart failure.

## Methods

### Study population

This study was conducted from March 2018 to May 2020.

Forty-three consecutive patients with non-diabetic, non-ischemic cardiomyopathy with CHF were enrolled in this study. Patients were eligible if mild to moderate CHF present ejection fraction (EF%) is between 30 and 40%, New York Heart Association Classification (NYHA class) is ranged between I and II, and age is between 40 and 80 years. Diabetes mellitus, known ischemic heart disease or previous CABG, active myocarditis, significant valve lesion, alcoholics, severe CHF (EF < 25%), NYHA class III and IV, patients in need for CCU admission, or patients that had been admitted to CCU within the last 3 months, patients who are fibrillating or with sustained ventricular tachycardia, patients with chronic kidney disease or with active liver disease alanine aminotransferase (ALT) > 3 folds, and patients with contraindication to B-blockers were exclusion criteria.

They were randomly assigned by a computer program. Patients were assigned to one of the two arms of the study either to receive carvedilol (carvedilol group *n* = 25( or nebivolol group; *n* = 18).

### Study protocol

After 2-weeks wash-out period (e.g., the patients received no B blocker treatment for a period of 2 weeks prior to their inclusion, to eliminate previous B blocker effect before starting carvedilol/nebivolol), our study protocol started including the following both at the beginning of the study as well as at the study end: NYHA class assessment, vitals (pulse/minute, blood pressure), body mass index (BMI), EF% (by Simpson Method), 6-minute walk test [15], routine laboratory investigations, fasting glucose level, glycosylated hemoglobin (HbA1C%), and fasting insulin of the blood samples collected for measurement of glucose and insulin. Plasma glucose was measured by glucose oxidase method with a Beckman glucose analyzer, and plasma insulin concentrations were determined by radioimmunoassay.

### Measurement of insulin sensitivity

The estimate of insulin resistance by homeostasis model of assessment (HOMA-IR) derives an estimate of insulin sensitivity from the mathematical modeling of fasting glucose and insulin concentrations [16]. In comparison to the euglycemic clamp, the HOMA-IR model is an easy, practical, and inexpensive method for assessing IR. We applied the HOMA-IR in non-diabetic participants using the following formula [17]: fasting insulin level (μU/ml) × fasting glucose (mg/dl)/405, subjects whose values exceeded the 75th percentile (i.e., 2.0) were considered to have insulin resistance (HOMA-IR index) [18].

### Measurement of norepinephrine

After 30 min rest, 9 ml of blood was drawn from an ante-cubital vein through the intravenous cannula, into pre-chilled tubes containing 15 ml EGTA (ethylene-glycol-tetra acetic-acid) and 12 mg glutathione. The tubes were kept on ice before and after blood sampling and were immediately centrifuged at 4 °C and 3000 rpm for 15 min, and then stored at − 80 °C until analysis by high-performance liquid chromatography [19]. All blood samples from the same patient were analyzed within the same setup.

### Measurement of plasminogen activator inhibitor (PAI-1)

Blood samples were collected on ice and centrifuged immediately at 0 °C for 20 min. All plasma or serum were separated and stored at − 80 °C until the time of assay. Blood for measurement of PAI-1 was collected in vacutainer tubes containing acidified 0.105 M sodium citrate (Becton Dickinson, Rutherford, NJ), as the use of anticoagulant minimizes the contribution of platelet activation to PAI-1 antigen concentrations. PAI-1 antigen level was determined using 2-site-enzyme-linked immunosorbent assays (Imulyse, Biopol AB).

The drug dose regimen for the carvedilol arm, a starting dose of 3.125 mg bid, was given; then, the dose was up-titrated to a target dose of 25 mg bid or the maximally tolerated dose for 12 weeks. For the nebivolol arm, a starting dose of 2.5 mg/day was given; then, the dose was up-titrated to a target dose of 10 mg/day or the maximally tolerated dose for 12 weeks. The further lines of treatment for heart failure were given according to the standard guidelines [20]. After 12 weeks clinical, laboratory follow-up, data were obtained through the outpatient department (OPD) visits.

The primary endpoint was evaluating the relative effects of those two B-blockers on insulin resistance as assessed by insulin resistance index) HOMA-IR) at the baseline and after 3 months treatment.

### Statistical analysis

The continuous variables were expressed in mean ± SD while discrete variables were expressed in percentage. The differences in continuous variables were checked for statistical significance by *t* test as appropriate; the differences in the discrete variables were checked for statistical significance by *X*^2^ test. The percentage of change at HOMA-IR between baselines versus at 3 months follow-up level of intragroup comparison was done. *P* value < 0.05 was considered significant. The statistical analysis was performed using SPSS.11 for Windows (SPSS Inc., Chicago, IL, USA).

## Results

Out of 43 patients enrolled, 58.1% represented the carvedilol group while 41.9% represented the nebivolol group. The demographic and clinical characteristics of our study population are shown in Table 1.

**Table 1:** Demographic, baseline clinical, and medications among the two groups

	Carvedilol group (***N*** = 25)	Nebivolol group (***N*** = 18)	***P*** value
Age	50.5 ± 10.5	50.9 ± 9.6	0.89
Wt (kg )	83.3 ± 12	81.4 ± 10	0.58
BMI(kg/m^2^)	37.0 ± 6.1	36.8 ± 4.3	0.9
Waist circum(cm )	118.6 ± 10.5	117.3 ± 9.9	0.68
Male gender	14 (56%)	10 (55.6%)	o.8
Smokers	8 (33%)	4 (22.2%)	0.48
TG (mg/dl)	132 ± 4.5	134 ± 3.5	0.12
HDL(mg/dl)	38 ± 6.2	39 ± 5.3	0.59
Uric acid(mg/dl)	8.1 ± 2.6	8.0 ± 2.3	0.89
Hb% (gm)	13.5 ± 1.2	13.0 ± 2.1	0.36
**Medications**			
Loop diuretic	17 (68%)	12 (66.7%)	0.93
Aldosterone blocker	15 (60%)	5 (27.8%)	0.036*
RAS blocker	23 (92%)	17 (94.4%)	0.99
Digoxin	6 (24%)	4 (22.2%)	0.99
ASA	19 (76%)	14 (77.8%)	0.99

*ASA* aspirin, *BMI* body mass index, *HB* hemoglobin, *HDL* high-density lipoprotein, *RAS* renin-angiotensin-system, *TG* triglyceride

*P is significant

No significant differences were found regarding age, weight, BMI, waist circumference, gender, triglyceride, high-density lipoprotein (HDL), uric acid, hemoglobin (HB) percentage, percentage of the use of loop diuretics, RAS blockers, digoxin, or aspirin. However, the study group showed significantly higher percentage of the use of aldosterone blockers among the carvedilol group (*P* = 0.036).

The intragroup versus intergroup comparison of variables at the baseline versus at 3-months follow-up is shown in Tables 2 and 3.

**Table 2:** Intra- versus intergroup comparison of variables at the baseline versus at 3 months follow-up among the two groups

		G1		G2		Inter-group	
		Baseline	After 3 months	Baseline	After 3 months	^c^*P*	^d^*P*
1. HR		102 ± 7	71 ± 5	103 ± 9	70 ± 6		
Intragroup *t* test		^a^*P* < 0.001*		^b^*P* < 0.001*		0.68	0.55
2. BP		137 ± 7	130 ± 8	135 ± 6	130 ± 9		
		85 ± 9	80 ± 10	32 ± 5	80 ± 6		
Intragroup *t* test		^a^*P* < 0.001*, < 0.001*		^b^*P* < 0.001*, < 0.02*		0.21	0.68
3. NYHA	Class I	20 (80%)	19 (76 %)	12 (66.7%)	14 (77.8%)		
	Class II	6 (24%)	6 (24%)	6 (33.3%)	4 (22.2%)		
Intragroup *t* test		^a^*P* = 0.03*		^b^*P* = 0.046*		0.85	0.99
4. EF%		39 ± 4.2	42 ± 3.0	38.9 ± 3.2	41.8 ± 3.3		
Intragroup *t* test		^a^*P* < 0.001*		^b^*P* < 0.001*		0.93	0.84
5. B blocker compliance		24 (96%)	24 (96%)	17 (94%)	17 (94%)		
Intragroup *t* test		^a^*P* > 0.99		^b^*P* > 0.99		0.99	0.99
6. HbA1c		5.8 ± 0.8	5.9 ± 0.3	5.9 ± 0.6	5.3 ± 0.2		
Intragroup *t* test		^a^*P* = 0.76		^b^*P* < 0.001*		0.55	< 0.001*
7. Fasting glucose (mg/dl)		102 ± 14	103 ± 9	103 ± 9	97 ± 7		
Intragroup *t* test		^a^*P* = 0.73		^b^*P* < 0.001*		0.31	0.023*
8. Plasma insulin (lU/ml)		5.3 ± 3.2	5.4 ± 3.9	5.4 ± 4.1	3.5 ± 0.23		
Intragroup *t* test		^a^*P* = 0.62		^b^*P* = 0.01*		0.92	0.046*
9. HOMA-IR		1.31 ± 0.29	1.37 ± 0.3	1.37 ± 0.3	1.21 ± 0.08		
Intragroup *t* test		^a^*P* = 0.41		^b^*P* = 0.01*		0.52	0.01*
10. Plasma norepinephrine (pg/ml%)		530 ± 159	436 ± 30	531 ± 171	499 ± 102		
Intragroup *t* test		^a^*P* = 0.002*		^b^*P* = 0.08		0.98	0.019*
11. Plasminogen activator I (ng/ml)		9.9 ± 3.2	9.8 ± 2.7	10.2 ± 2.5	10.1 ± 3.3		
Intragroup *t* test		^a^*P* = 0.37		^b^*P* = 0.81		0.74	0.75

*HR* heart rate, *BP* blood pressure, *HbA1c* glycated hemoglobin, *HOMA-IR* homeostasis model of assessment, *NYHA* New York Heart Association Classification, *EF* ejection fraction, *G1* group 1, *G2* group 2

*P is significant

^a^*P* intragroup comparison of group 1

^b^*P* intragroup comparison of group 2

^c^*P* intragroup comparison of group 1; intergroup comparison of group 1 and group 2 at baseline

^d^*P* intergroup comparison of group 1 and group 2 after 3 months

**Table 3:** Intra- versus intergroup comparison of 6 min walk test at the baseline versus at 3 months follow-up for the two groups

	G1		G2		Intergroup	
	Baseline	After 3 months	Baseline	After 3 months	^c^*P*	^d^*P*
**A. Mean distance walked (m)**						
	322 ± 104	404 ± 108	326 ± 100	407 ± 104.0		
	^a^*P* < 0.001*		^b^*P* < 0.001*		0.9	0.93
**B. No. of patients walked < 300 m**						
	9 (36%)	3 (12%)	7 (38.9%)	2 (11.1%)		
Intragroup *t* test	^a^*P* = 0.031*		^b^*P* = 0.063*		0.85	0.99

*P is significant

^a^*P*, intragroup comparison of group 1

^b^*P*, intragroup comparison of group 2

^c^*P*, intragroup comparison of group 1; intergroup comparison of group 1 and group 2 at baseline

^d^*P*, intergroup comparison of group 1 and group 2 after 3 months

No significant differences were found neither on intragroup nor on intergroup comparison level (both at the baseline and at follow-up) regarding B blocker compliance or plasminogen activator inhibitor level. Likewise, no significant differences were found on intergroup comparison level (both at the baseline and at follow-up) regarding heart rate, blood pressure, NYHA class, EF%, or 6 min walk test.

**Fig. 1:**
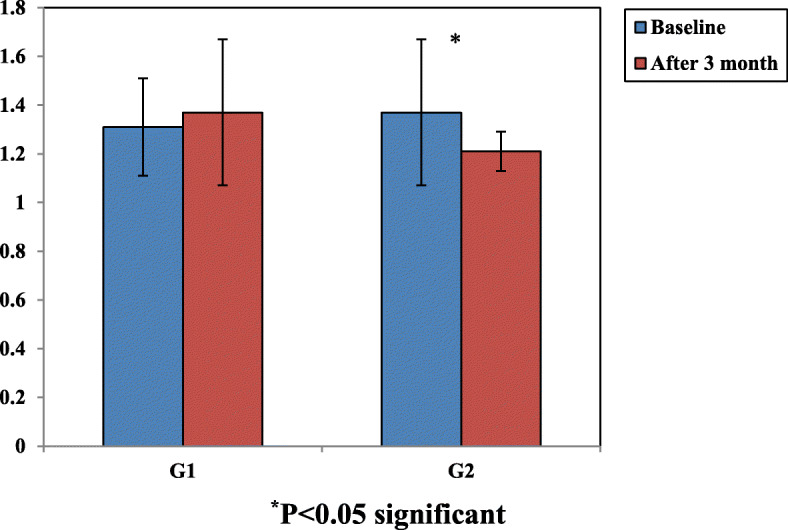
Homeostatic model of assessment for insulin resistance in both groups. X-axis refers to the studied groups, Y-axis refers to the HOMA-IR value; G1, group 1; G2, group 2

The fasting glucose, fasting insulin, and HOMA-IR showed no significant difference on intragroup comparison (for carvedilol group only) and on intergroup comparison at baseline (for the two groups); however, these 3 variables were significantly lower among the nebivolol group (at 3 months follow-up only) both on intragroup and intergroup comparison (*P* < 0.001, 0.01, and 0.01) respectively (HOMA-IR changes are shown in Fig. 1).

The norepinephrine level was significantly lower in the carvedilol group (G1) on intragroup level of comparison on 3 months follow-up (*P* = 0.002). On the other hand, its level showed no significant difference at the nebivolol group (G2) neither on intragroup nor on intergroup comparison level with the nebivolol group (G2).

The PA-I level showed non-significant difference in both carvedilol group (G1) and nebivolol group (G2) whether on intragroup or intergroup level of comparison.

The following variables were significantly improved (at 3 months follow-up) on intragroup level of comparison, e.g., NYHA class, EF%, mean distance walked, and number of patients walked < 300 m (*P* = 0.03, *P* = 0.046, *P* < 0.001, *P* < 0.001, *P* < 0.001, *P* < 0.001, *P* = 0.031, and *P* = 0.063) respectively. Likewise, blood pressure and heart rate were also significantly decreased on intragroup level of comparison (*P* < 0.001, *P* < 0.001, *P* < 0.001, < 0.02, *P* < 0.001, < 0.001) respectively. The percentage of change at HOMA-IR, indicative of insulin sensitivity status, between baseline versus at 3 months follow-up level of intragroup comparison is shown in Table 4. The percentage of change was increased by 4.58% at the carvedilol arm whereas was decreased by 11.67% at the nebivolol arm, and the difference on the intra-group level of comparison was significant (*P* < 0.001 and 0.01) respectively.

**Table 4:** Comparison between both groups

	Studied groups			
	Carvedilol group (*n* = 25)		Nebivolol group (*n* = 18)	
	Before	After	Before	After
HOMA-IR	1.31 ± 0.29	1.37 ± 0.3	1.37 ± 0.3	1.21 ± 0.08
^*P*	***P*** **< 0.001**		***P*** **= 0.01**	
Percent of change	Increase (4.58%)		Decreased (11.67%)	
Norepinephrine	530 ± 159	436 ± 30	531 ± 171	499±102
^*P*	***P*** **< 0.002**		***P*** **= 0.08**	
Percent of change	Decreased ( 17.73%)		Decreased (6.02%)	

*HOMA-IR* homeostasis model of assessment

^Paired *t* test

*P is significant

## Discussion

The interrelationship between HF and IR, plasma norepinephrine, and B-blockers was discussed above [2–11, 14].

Results of our study demonstrated that nebivolol but not carvedilol improves insulin sensitivity while carvedilol but not nebivolol decreases plasma norepinephrine level. Our results of favorable effect on nebivolol on insulin sensitivity in HF patients can be explained by Manrique and colleagues [21]; they conducted an experimental study on insulin resistance Sprague-Dawley rat model treated with nebivolol for 3 weeks; they assessed HOMA-IR index as well as nicotinamide adenine dinucleotide phosphate (NADPH) oxidase activity (NADPH is an insulin metabolic signaling in skeletal muscle) before and after nebivolol treatment. They concluded that treatment with nebivolol was associated with improvement in insulin resistance with decreased NADPH oxidase activity level. A previous study [22] demonstrated that the improvement in insulin sensitivity is closely associated with decreased NADPH oxidase activity in skeletal muscle.

Previous clinical trials had investigated the value of B-blockers in CHF [23–25].

One study [23] conducted a prospective double-blinded, placebo-controlled randomized study on 46 CHF patients who received carvedilol or placebo to investigate whether treatment with carvedilol alter insulin sensitivity or not. They found that neither insulin sensitivity nor plasma norepinephrine had been significantly altered. Differently from this study, the present study demonstrated a significant decrease in the plasma norepinephrine level among the carvedilol group.

Ferrua et al. [24] reported that carvedilol significantly reduced HOMA-index in non-diabetic CHF patients; this was contradictory to our results.

Another pilot study investigated the nebivolol effect in CHF patients reported; after 6 weeks period of treatment, plasma catecholamine remained unchanged at rest and during exercise [25]. Ayers et al. [26] reported that nebivolol has a neutral effect on IR in metabolic syndrome (Met S) patients. Apart from studies on carvedilol and nebivolol, De Groote and colleagues [27] investigated 3 months bisoprolol effect in patients with stable congestive heart failure; they reported EF% improvement with a significant decrease in plasma norepinephrine level.

The decrease of plasma norepinephrine with carvedilol found in our study could be explained by a sympathoinhibitory action through blocking peripheral B receptor [28]. Grundemar et al. [29] reported that carvedilol blocks B1, B2, and α1 adrenergic receptors at a higher dose. Also, it does not modulate B1 receptor (e.g., no upregulation or downregulation), thus, exerting much anti adrenergic properties compared to the selective B-blockers.

Although there was a significantly higher use of aldosterone antagonist (a drug known to improve insulin resistance in patients with chronic heart failure [30]) among the carvedilol group, however, this did not attenuate carvedilol effect on IR (e.g., carvedilol takes the upper hand).

Our result demonstrated normal serum PAI-1 level, with no significant differences between the two groups of our study population. According to some recent studies, the relation between PAI-1 and the Met S criteria needed for diagnosis may not always be straight forward and needs more study [31].

## Conclusion

Nebivolol improved the insulin resistance-related variables (fasting glucose, fasting insulin, and HOMA-IR) while carvedilol was neutral. Meanwhile, carvedilol decreased plasma norepinephrine level while nebivolol was neutral. Lastly, both B-blockers improved the hemodynamics-related variables by the same extent. Nebivolol may be recommended as the B blocker of the first choice for non-ischemic cardiomyopathy heart failure patients complicated by insulin resistance; however, larger scaled prospective multicenter randomized trials are needed for confirming our favorable results.

## Study limitations

Firstly, our sample size was relatively small; a larger scaled prospective multicenter randomized trial is needed. Secondly, long-term follow-ups are not studied.

## Data Availability

All data and material of the research are available if requested.

## Abbreviations

ALT: Alanine aminotransferase
BMI: Body mass index
CABG: Coronary artery bypass grafting
CHF: Congestive heart failure
RAS blocker: Renin-angiotensin system blocker
EGTA: Ethylene-glycol-tetra acetic-acid
EF: Ejection fraction
HB: Heart block
HFrEF: Heart failure with reduced ejection fraction
HDL: High-density lipoprotein
HOMA-IR: Homeostasis model of assessment
HbA1c: Glycosylated hemoglobin
IR: Insulin resistance
NE: Norepinephrine
NADPH: Nicotinamide adenine dinucleotide phosphate
NYHA: New York Heart Association Classification
PAI: Plasminogen activator inhibitor
OPD: Outpatient department
SNS: Sympathetic nervous system
